# Communicating carotid-cavernous sinus fistula following minor head trauma

**DOI:** 10.1186/1865-1380-5-10

**Published:** 2012-02-13

**Authors:** Joshua B Kaplan, Aakash N Bodhit, Michael L Falgiani

**Affiliations:** 1 Department of Emergency Medicine, University of Florida College of Medicine, 1329 SW 16th Street, PO Box 100186, Gainesville, FL 32610, USA

## Abstract

**Introduction:**

A case of communicating carotid-cavernous sinus fistula (CCF) after minor closed head injury is presented.

**Case presentation:**

A 45-year-old Caucasian male presented to the emergency department of a tertiary care hospital with the chief complaint of blurred vision and facial numbness. The patient had experienced a minor head injury 1 month ago with loss of consciousness. After a 2-week symptom-free period, he developed scalp and facial numbness, along with headache and vision problems. His vital signs were within normal limits, but on examination the patient was noted to have orbital and carotid bruits with several concerning neurological findings. CT and MRI confirmed the suspicion of carotid-cavernous sinus fistula, which was managed by cerebral angiography with coil embolization of this fistula. The patient was symptom free at the 8-month follow-up.

**Discussion:**

Carotid-cavernous sinus fistula is a rare condition that is usually caused by blunt or penetrating trauma to the head, but can develop spontaneously in about one fourth of patients with CCF. The connection between the carotid artery and cavernous sinus leads to increased pressure in the cavernous sinus and compression of its contents, and thereby produces the clinical symptoms and signs seen. Diagnosis depends on clinical examination and neuroimaging techniques. The aim of management is to reduce the pressure within the cavernous sinus, which results in gradual resolution of symptoms.

## Introduction

A carotid-cavernous sinus fistula (CCF) is a rare condition well known in the neurosurgical field, but not well published in emergency medicine literature. Whether post-traumatic or spontaneous in nature, the symptoms related to CCFs are insidious and potentially severe. From chronic headaches and diplopia, to intracranial hemorrhage [1] and permanent vision loss [2,3], the ability to identify and manage this disease is essential. The clinical presentations of CCFs can be varied as well, mimicking diseases like multiple sclerosis, brain tumors, or stroke, making it imperative that emergency physicians in the right setting include this disease in their differential diagnosis. We report a case of a patient with a communicating CCF that presented to our Emergency Department (ED) 1 month after suffering a closed head injury at a party.

## Case presentation

A 45-year-old Caucasian male presented to the emergency department with the chief complaint of "blurred vision and facial numbness." The patient recalled that a month ago he had been drinking with friends when he was either punched or struck in the back of the head by an unknown object. He lost consciousness but did not seek medical attention at the time, stating that he "felt fine." He remained symptom-free for the next 2 weeks, until he developed left scalp and left facial numbness, and noted that he could not clench his jaw tightly on that side.

Over the next week his symptoms had increased to include: right-sided scalp and forehead numbness, right-sided droopy eyelid, light sensitivity, double vision, difficulty walking in a straight line, bilateral pulsating tinnitus, and a throbbing occipital to retro-orbital headache. With increasing difficulty performing his job as an electrician, he presented to the ED seeking medical attention for the first time.

His past medical history was significant for alcoholism and an old frontal bone fracture suffered as a child. He was not on any medications and had no significant family history. Comprehensive review of systems was otherwise noncontributory.

On physical examination, the patient was wearing sunglasses, in no acute distress. Vital signs were unremarkable. He appeared clinically sober. His visual acuity was 20/30 in each eye, and there were bilateral orbital bruits. The patient declined fundoscopic examination because of significant photophobia. The right eye exhibited: ptosis, inability to adduct, limited elevation and depression. He also could not abduct his left eye. He had decreased sensation to soft touch over the entire forehead, nose, and left cheek, but his corneal reflexes were intact. There was no facial asymmetry. The rest of the cranial nerve examination was intact. Motor strength bilaterally did not reveal any focal neurological deficits, and deep tendon reflexes (DTRs) were 2/4 symmetrical in both the upper and lower extremities. The cerebellar examination was unremarkable except for abnormal tandem gait. Other systemic examinations were unremarkable except for bilateral carotid bruits.

**Figure 1:**
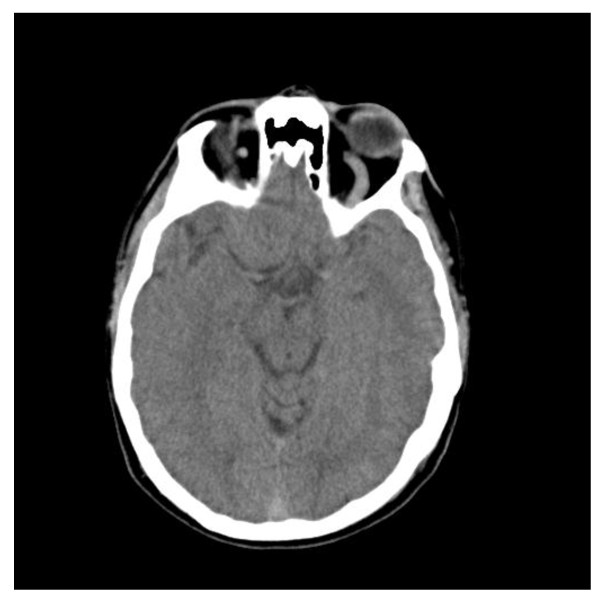
**CT of the head without contrast**. Left ophthalmic vein is dilated.

The initial workup in the ED included a complete blood count (CBC), basic metabolic panel (BMP), coagulation studies [prothrombin time (PT), partial thromboplastin time (PTT), international normalization ratio (INR)], and a urine toxicology screen. Each of these studies was without abnormality. A computed tomography (CT) scan of the head without contrast was performed, showing a significantly dilated left ophthalmic vein, seen in Figure 1.

**Figure 2:**
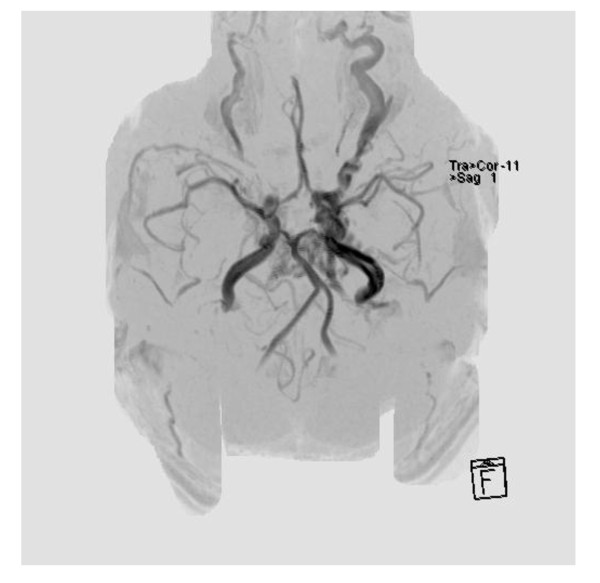
**MRI of the brain with contrast**. Increased flow in the left cavernous sinus and engorgement of the left ophthalmic vein.

**Figure 3:**
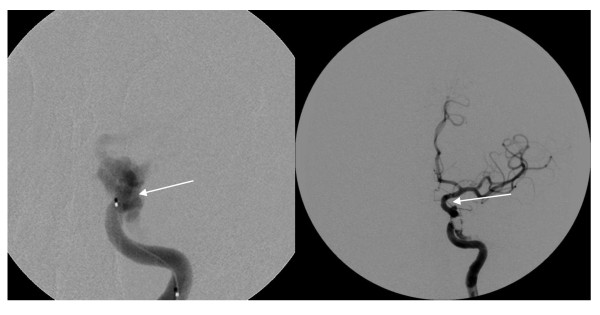
**Cerebral angiography images**. (*Left*) Angio-catheterization of the left internal carotid artery (*ICA*) showing a CCF (*arrow*). (*Right*) Post-coil embolization (*arrow*) of the left CCF.

Subsequent brain magnetic resonance imaging (MRI) confirmed the suspicion of a carotid artery-cavernous sinus fistula (Figure 2). Neurosurgery was consulted. The following day, cerebral angiography with coil embolization of the carotid fistula was performed without complication (Figure 3). The patient was discharged the next day. An 8-month follow-up in the neurosurgery clinic revealed complete resolution of his symptoms.

## Discussion

We report a rare condition encountered in the emergency department of a post-traumatic carotid-cavernous fistula. CCFs are formed when there is leakage of arterial flow from the carotid artery into the venous system of the cavernous sinus. This pathological connection leads to rising pressure within the cavernous sinus and compression of its contents, including cranial nerves (CN) III, IV, V, and VI. Involvement of these nerves results in ophthalmoplegia, facial sensory deficits, ptosis, and photophobia. Mounting venous hypertension causes engorgement of the ophthalmic vessels leading to the classic triad of CCFs: orbital bruit, chemosis, and pulsating exophthalmos [4-7]. Pressure can also be transmitted to the contralateral cavernous sinus via the presence of intercavernous bridging vessels [8], resulting in bilateral CN deficits and ocular findings, as is seen in this case report.

The vast majority of CCFs follow blunt or penetrating trauma to the head [7,9,10], with only one quarter occurring spontaneously (often from ruptured aneurysms) [11]. Most cases described in the literature are secondary to closed head injuries related to motor vehicle accidents [7,12], but as seen in this case, any type of closed head injury can result in a CCF. Symptom onset is typically delayed until venous hypertension reaches a critical level, often days to weeks following the initial insult.

CCFs can be categorized using the Barrow Classification System. Type A fistulas involve a direct communication between the intercavernous portion of the internal carotid artery (ICA) and the cavernous sinus, whereas types B, C, and D are indirect communications between either dural branches of the ICA or external carotid artery (ECA) and the cavernous sinus [7,13]. Type A CCFs are common in young males, as this demographic has a higher incidence of closed head injuries, whereas indirect CCFs occur more often in the elderly [13].

It is important to distinguish between direct (type A) and indirect (types B-D) fistulas because of the prognostic implications. Direct CCFs are typically high flow and result in significant venous hypertension, while indirect fistulas tend to be low flow [7,14]. These low-flow lesions generally have fewer and less severe symptoms [7], they improve with time, and often can be medically managed [1,15]. In contrast, it is recommended that all high-flow lesions receive surgical treatment as these can progress to intracranial hemorrhage [1], vision loss [2,3], and even life-threatening epistaxis [16].

The diagnosis of CCFs is based on clinical presentation as well as neuroimaging. A CT scan of the head without contrast may show proptosis, engorgement, and tortuosity of the superior ophthalmic vein, and enlargement of the affected cavernous sinus. MRI images will typically show similar but more pronounced findings compared to CT, and are particularly useful in classifying CCFs [7,13]. If a direct, high-flow CCF is identified, the treatment of choice is endovascular embolization [13,14]. Once the lesion has been embolized, pressure within the cavernous sinus will normalize and symptoms will begin to resolve.

## Abbreviations

CCF: Carotid-cavernous sinus fistula; ED: emergency department; DTR: deep tendon reflexes; CBC: complete blood count; BMP: basic metabolic panel; PT: prothrombin time; PTT: partial thromboplastin time; INR: international normalized ratio; CT: computed tomography; MRI: magnetic resonance imaging; CN: cranial nerves; ICA: internal carotid artery; ECA: external carotid artery.

## Consent

The patient has given consent to present the case and for the use of images of diagnostic procedures.

## Competing interests

The authors declare that they have no competing interests.

## Authors' contributions

JBK and MLF did the clinical examination and clinically managed the patient. JBK drafted the case report manuscript. ANB edited and formatted the manuscript and drafted the abstract for the case report, and prepared the final draft for submission. All authors read and approved the final manuscript.

## Authors' information

JBK is the Chief Resident Physician in the Department of Emergency Medicine, University of Florida College of Medicine at Gainesville, FL. ANB is the Research Fellow/Coordinator in the Department of Emergency Medicine, University of Florida. MLF is the Clinical Assistant Professor at the Department of Emergency Medicine, University of Florida College of Medicine, Gainesville, Florida.

## References

[B1] Halbach VV Higashida RT Hieshima GB Dural fistulas involving the cavernous sinus: results of treatment in 30 patients Radiology 1987 163 437 442 3562823 10.1148/radiology.163.2.3562823

[B2] Vinuela F Fox AJ Debrun GM Spontaneous carotid-cavernous fistulas: clinical, radiological, and therapeutic considerations: experience with 20 cases J Neurosurg 1984 60 976 984 10.3171/jns.1984.60.5.0976 6716167

[B3] Halbach VV Hieshima GB Higashida RT Carotid cavernous fistulae: indications for urgent treatment Am J Roentgenol 1987 149 587 593 10.2214/ajr.149.3.587 3497549

[B4] Lewis AI Tomsick TA Tew JM Jr Management of 100 consecutive direct carotid-cavernous fistulas: results of treatment with detachable balloons Neurosurgery 1995 36 239 245 10.1227/00006123-199502000-00001 7731502

[B5] Lewis AI Tomsick TA Tew JM Jr Lawless MA Long-term results in direct carotid-cavernous fistulas after treatment with detachable balloons J Neurosurgery 1996 84 400 404 10.3171/jns.1996.84.3.0400 8609550

[B6] DeBrun G Lacour P Vinuela F Treatment of 54 traumatic carotid-cavernous fistulas J Neurosurg 1981 55 678 692 10.3171/jns.1981.55.5.0678 6458669

[B7] Chaudry AI Carotid cavernous fistula: ophthalmological implications Middle East Afr J Ophthalmology 2009 16 2 57 63 10.4103/0974-9233.53862 PMC2813585 20142962

[B8] Aquini M Intercavernous venous communications in the human skull base Skull Base Surgery 1994 4 3 145 150 10.1055/s-2008-1058966 17171164 PMC1661802

[B9] Abrahamson IA Jr Bell LB Jr Carotid-cavernous fistula syndrome Am J Ophthalmol 1955 39 521 526 14361579 10.1016/0002-9394(55)92249-2

[B10] Keltner JL Satterfield D Dublin AB Dural and carotid cavernous sinus fistulas: Diagnosis, management, and complications Ophthalmology 1987 94 1585 1600 3323984 10.1016/s0161-6420(87)33258-0

[B11] Barrow DL Spector RH Braun IF Classification and treatment of spontaneous carotid cavernous sinus fistulas J Neurosurg 1985 62 248 256 10.3171/jns.1985.62.2.0248 3968564

[B12] Locke CE Intracranial arteriovenous aneurism or pulsating exophthalmos Ann Surg 1924 80 1 24 17865057 10.1097/00000658-192480010-00001 PMC1399672

[B13] Gemmete J Chaudhary N Pandey A Treatment of carotid cavernous fistulas Current Treatment Options in Neurology 2010 12 43 53 10.1007/s11940-009-0051-3 20842489

[B14] Corradino G Gellad FE Salcman M Traumatic carotid cavernous fistula South Med J 1988 81 660 663 10.1097/00007611-198805000-00030 3368818

[B15] Higashida RT Hieshima GB Halbach VV Closure of carotid cavernous sinus fistulae by external compression of the carotid artery and jugular vein Acta Radiol Suppl 1986 369 580 583 2980563

[B16] Jiamsripong P Mookadam M Mookadam F An uncommon cause of epistaxis: carotid cavernous fistula Emerg Med J 2007 24 e28 10.1136/emj.2006.045195 17452688 PMC2658512

